# Supplementary material for: In Vitro and In Vivo Effect of the Imidazole Luliconazole against Lomentospora prolificans and Scedosporium spp
Source: Microbiol Spectr. 2023 Apr 5;11(3):e05130-22. doi: 10.1128/spectrum.05130-22 (PMC10269907; doi:10.1128/spectrum.05130-22)
Supplement: Supplemental file 1 — Supplemental material. Download spectrum.05130-22-s0001.pdf, PDF file, 0.9 MB [file spectrum.05130-22-s0001.pdf]

Appendix (Supplemental figures)

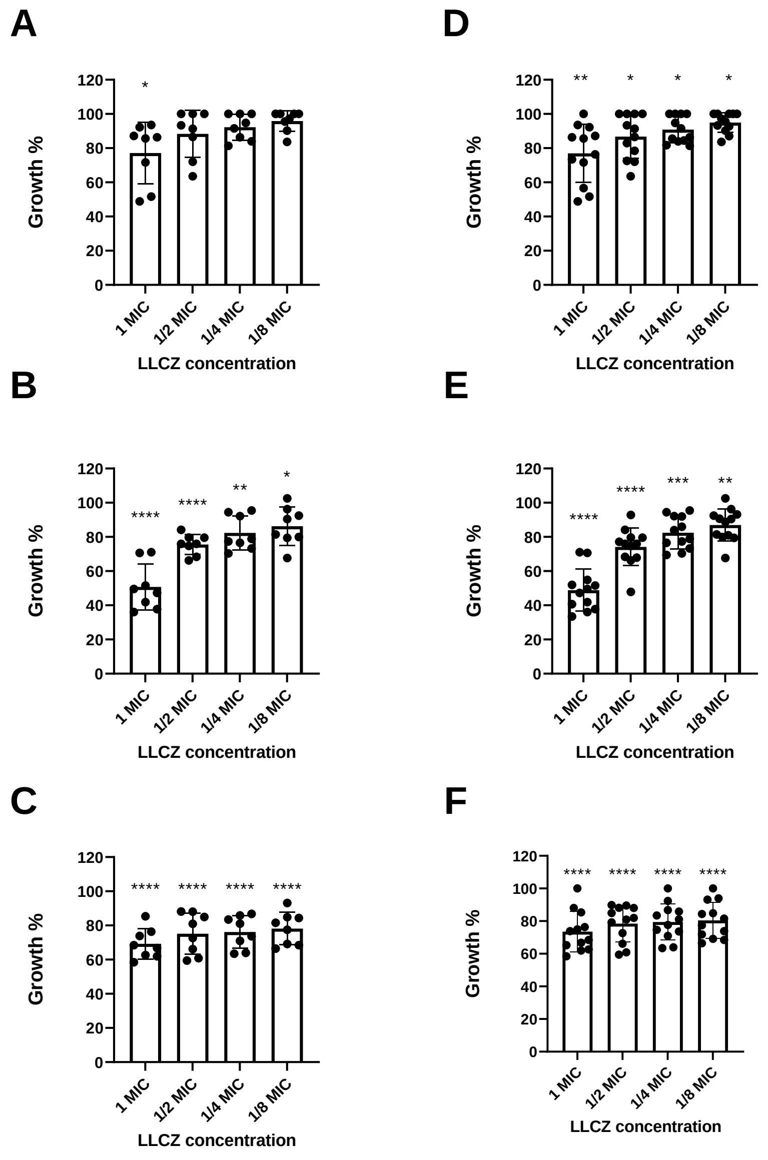

Figure A1. A-C: Growth of *L. prolificans* (8 isolates) biofilm; **D-F**: growth of all organisms (8 isolates *L. prolificans*, 6 isolates *S. apiospermum*/ *P. boydii*), when treated with LLCZ (CV assay). Isolates were treated with concentrations of 1-, 1/2-, 1/4- and 1/8 times the isolate specific minimal inhibitory concentration at: 0 h, 2 h and 48 h after incubation. Statistical significance was determined by Dunnett's multiple comparison tests. *: $p < 0.05$; **: $p < 0.01$; ***: $p < 0.001$; ****: $p < 0.0001$.

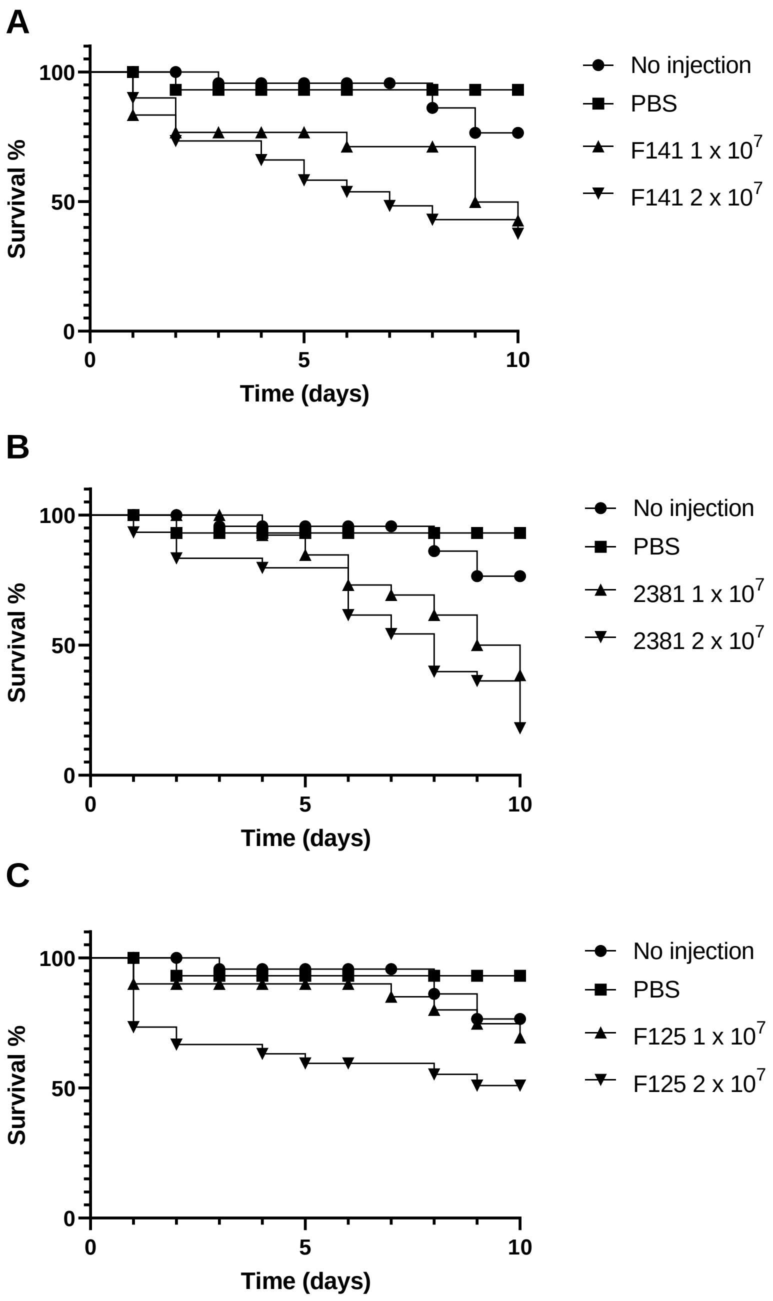

Figure A2. Survival curves for *Galleria mellonella* larvae infected with: **A**. *L. prolificans* (isolate F141 – reference strain), **B**. *S. apiospermum* (isolate 2381 – patient isolate) and **C**. *P. boydii* (isolate F125 – reference strain). Groups of 15 larvae were injected once with a fungal inoculum of 1 x 10^7 CFU/ml and 2 x 10^7 CFU/ml, while control groups were injected with PBS or not injected at all, in order to rule out any false positive results caused by the piercing damage of the needle. The volume injected was dependent on the weight of the larvae (10-16.66 µl). The larvae were incubated at 37 °C and mortality was checked every 24 h for at least 10 days.
